# Expanding Diagnostic Options for Pediatric Meningitis: BCID2 Testing Results on Cerebrospinal Fluid After a Negative Meningitis/Encephalitis Panel

**DOI:** 10.3390/antibiotics15050519

**Published:** 2026-05-21

**Authors:** Venere Cortazzo, Lorenza Romani, Gianluca Vrenna, Maia De Luca, Marilena Agosta, Martina Rossitto, Valeria Fox, Barbara Lucignano, Manuela Onori, Stefania Mercadante, Vito Tommaso, Laura Lancella, Stefania Bernardi, Mara Pisani, Alessandra Salvatori, Alberto Villani, Massimiliano Raponi, Carlo Federico Perno, Paola Bernaschi

**Affiliations:** 1 Microbiology and Diagnostic Immunology Unit, Bambino Gesù Children’s Hospital, IRCCS, 00165 Rome, Italy; venere.cortazzo@opbg.net (V.C.); marilena.agosta@opbg.net (M.A.); barbara.lucignano@opbg.net (B.L.); manuela.onori@opbg.net (M.O.); vito.tommaso@opbg.net (V.T.); carlofederico.perno@opbg.net (C.F.P.); paola.bernaschi@opbg.net (P.B.); 2 Infectious Disease Unit, Bambino Gesù Children’s Hospital, IRCCS, 00165 Rome, Italy; lorenza.romani@opbg.net (L.R.); maia.deluca@opbg.net (M.D.L.); stefania.mercadante@opbg.net (S.M.); laura.lancella@opbg.net (L.L.); stefania.bernardi@opbg.net (S.B.); 3 Multimodal Laboratory Medicine, Bambino Gesù Children’s Hospital, IRCCS, 00165 Rome, Italy; martina.rossitto@opbg.net (M.R.); valeria.fox@opbg.net (V.F.); 4 Paediatrics Clinical Unit, Bambino Gesù Children’s Hospital, IRCCS, 00165 Rome, Italy; mara.pisani@opbg.net (M.P.); alessandra.salvatori@opbg.net (A.S.); alberto.villani@opbg.net (A.V.); 5 Medical Direction, Bambino Gesù Children’s Hospital, IRCCS, 00165 Rome, Italy; massimiliano.raponi@opbg.net

**Keywords:** pediatric meningitis, BioFire FilmArray BCID2, cerebrospinal fluid, molecular diagnostics, off-label use, multidrug-resistant organisms, antimicrobial stewardship

## Abstract

Background: Rapid etiological diagnosis of bacterial meningitis is crucial in children, as delays can lead to neurological sequelae. The BioFire FilmArray Meningitis/Encephalitis (ME) panel is widely used on cerebrospinal fluid (CSF), but its target spectrum may miss healthcare-associated or multidrug-resistant pathogens. We evaluated the diagnostic performance and stewardship-oriented clinical impact of off-label BioFire FilmArray Blood Culture Identification 2 (BCID2) testing on CSF from pediatric patients with suspected bacterial CNS infection and negative ME results. Methods: We retrospectively analyzed CSF samples collected between January 2023 and March 2025 at a tertiary pediatric hospital. In ME-negative cases with persistent suspicion and abnormal CSF parameters, BCID2 was performed off-label on residual CSF aliquots after routine testing, without additional sampling. We assessed pathogen detection, agreement with culture, resistance-gene identification, and documented stewardship actions. Results: Among 76 ME-negative CSF samples tested with BCID2, 23 (30.3%) were positive, all involving organisms not included in the ME panel. BCID2 was concordant with culture in 19/23 cases (82.6%); 4/23 (17.4%) were BCID2-positive/culture-negative, consistent with reduced culture sensitivity in frequently pretreated cases. Resistance genes (VIM, vanA/B, CTX-M) were detected in 30.4% of BCID2-positive samples. Overall agreement with culture was 94.7% (PPA 100%, NPA 93.0%). Escalation was documented in 13/23 episodes (56.5%), discontinuation in 2/23 (8.7%), and confirmation in 9/23 (39.1%), with no de-escalation events; clinical outcomes were not systematically available. Conclusions: In selected ME-negative pediatric cases with abnormal CSF profiles, BCID2 testing on residual CSF provided rapid, clinically meaningful microbiological information that may support antimicrobial optimization.

## 1. Introduction

Bacterial meningitis remains a life-threatening medical emergency that requires rapid diagnosis and timely initiation of appropriate antimicrobial therapy. Despite advances in clinical management, it continues to be associated with substantial morbidity and mortality, particularly in neonatal and pediatric populations. Delays in diagnosis or incomplete identification of the causative pathogen may result in irreversible neurological injury, hearing/sight loss, or long-term cognitive impairment. Globally, bacterial meningitis is associated with substantial case fatality, and up to one in five survivors may experience long-term disability, with the highest burden in infants and children [1,2,3].

Conventional diagnostic approaches, including cerebrospinal fluid (CSF) Gram stain and culture, are limited by prolonged turnaround times and reduced sensitivity. These limitations are especially evident in patients who have received empirical antimicrobial therapy prior to lumbar puncture, as well as in infections caused by fastidious or slow-growing organisms. In this context, multiplex molecular syndromic panels have significantly reshaped the diagnostic landscape by enabling rapid detection of multiple pathogens directly from CSF, using minimal sample volumes and without the need for viable microorganisms.

The BioFire^®^ FilmArray^®^ Meningitis/Encephalitis (ME) panel is approved for CSF testing and has demonstrated high sensitivity and specificity across clinical studies and meta-analyses [4,5]. However, its diagnostic scope is restricted to a predefined panel of common pathogens and does not include some clinically relevant pathogens, such as multidrug-resistant Gram-negative bacilli and fungal pathogens, which are increasingly encountered in healthcare-associated infections and in immunocompromised patients.

When the ME panel yields negative results despite persistent clinical suspicion of bacterial meningitis, additional diagnostic strategies may be required. The BioFire^®^ FilmArray^®^ Blood Culture Identification 2 (BCID2) panel, although validated only for use on positive blood culture bottles, offers substantially broader microbiological coverage. In addition to a wide range of Gram-positive and Gram-negative bacteria and yeasts, it includes molecular detection of key antimicrobial resistance genes, providing early insight into resistance mechanisms that may influence therapeutic decisions. This is particularly relevant in healthcare-associated or device-related CNS infections, where organisms such as staphylococci, enterococci, non-fermenting Gram-negative bacilli, and Candida spp. may occur and resistance information can help optimize early empiric coverage.

Although the use of BCID2 on CSF is considered off-label, its application has been described in selected clinical reports and small studies involving sterile body fluids, including CSF and synovial fluid, with encouraging diagnostic performance [6,7,8,9]. In high-risk pediatric populations, such as pretreated, critically ill, or immunocompromised patients, where conventional methods frequently fail to identify the causative pathogen, the expanded diagnostic spectrum of BCID2 may provide clinically meaningful information within a few hours.

Compared with conventional culture, which typically requires 24 to 72 h for organism identification and antimicrobial susceptibility testing, BCID2 yields results within approximately 1.5 to 2 h. This marked reduction in turnaround time has the potential to support earlier, more informed antimicrobial decision-making, particularly in critically ill children, or patients at risk of CSF shunt infection, for whom diagnostic is challenging and a delay may adversely affect outcomes.

Although prospective validation of BCID2 on CSF is currently lacking, available off-label evidence suggests that it may complement existing molecular diagnostic tools in carefully selected clinical scenarios, particularly when rapid microbiological information is needed to guide patient management.

In this retrospective study, we evaluated the off-label application of the BCID2 panel on CSF samples from neonatal and pediatric patients with suspected bacterial meningitis admitted to Bambino Gesù Children’s Hospital (IRCCS), Rome, between January 2023 and March 2025. The primary objectives were to characterize the spectrum of pathogens detected by BCID2, assess its concordance with conventional culture, and explore its potential clinical impact in terms of diagnostic timeliness and antimicrobial stewardship.

Given the limitations of standard culture-based diagnostics (whose turnaround time is not fully consistent with the clinical needs of a rapid and effective antimicrobial therapy) and the ME panel, especially in pretreated, immunocompromised patients, or CSF shunt carriers, we hypothesized that BCID2 could function as a valuable adjunctive diagnostic tool, enabling rapid identification of clinically significant pathogens and resistance markers, including in selected culture-negative cases.

## 2. Results

Between January 2023 and March 2025, a total of 689 CSF samples were processed at Bambino Gesù Children’s Hospital (Rome, Italy) using conventional culture and the ME panel. Of these, 129 samples (18.7%) tested positive on the ME panel, while 560 (81.3%) were ME-negative. Among the ME-negative samples, 76 CSF specimens underwent off-label BCID2 testing in real time (using residual CSF) in clinically selected cases with persistent suspicion of CNS infection and abnormal CSF chemical–physical profiles. Eligible episodes were subsequently identified and reviewed retrospectively for the purposes of this study. An overview of diagnostic positivity and concordance is reported in Table 1.

Of the 76 ME-negative CSF samples tested with the BCID2 panel, 23 (30.3%) yielded a positive result, corresponding to 19 individual patients. The most frequently detected pathogens were *Staphylococcus* spp. (*n* = 5), *Candida albicans* (*n* = 5), *Pseudomonas aeruginosa* (*n* = 4), *Enterococcus faecium* (*n* = 3), *Streptococcus pyogenes* (*n* = 2), *Escherichia coli* (*n* = 2), *Candida parapsilosis* (*n* = 1), and *Salmonella* spp. (*n* = 1). Two patients were craniopagus twins with the same clinical syndrome and identical *C. albicans* detection; they were retained as separate diagnostic episodes because they represented two distinct patients and two independently tested CSF samples. However, for pathogen-frequency interpretation, they may also be considered as a single epidemiologically linked event, in which case *C. albicans* accounts for 4/22 events rather than 5/23 diagnostic episodes, without materially changing the overall pathogen distribution. Clinically relevant antimicrobial resistance genes were identified in 7 of the 23 BCID2-positive samples (30.4%), including VIM (*n* = 4), *van*A/B (*n* = 2), and CTX-M (*n* = 1). None of these pathogens are present in the ME panel.

BCID2 was negative in 53/76 samples (69.7%); all 53 were also culture negative. Most patients had received empirical antimicrobial therapy prior to CSF sampling. BCID2 results were available within hours, whereas culture-based organism identification required longer and was variable across cases (typically within 24–72 h), and final culture reporting could take several days. In 19 of the 23 BCID2-positive samples (82.6%), molecular findings were concordant with conventional culture. In the remaining four cases (17.4%), BCID2 was positive while culture turned to be negative. The four discordant episodes involved *Streptococcus* spp., *Streptococcus pyogenes*, *Staphylococcus aureus*, and *Enterococcus faecium*. In the *Streptococcus* spp. and *S. aureus* episodes, other clinical specimens collected on the same day yielded concordant organisms (deep wound pus culture positive for *Streptococcus intermedius*; pus and biopsy cultures positive for *S. aureus*, respectively), lending support to the clinical plausibility of these molecular findings. No concomitant microbiological positivity from other anatomical sites was documented for the *S. pyogenes* and *E. faecium* episodes. Notably, these discordant episodes occurred while patients were receiving antimicrobial therapy at the time of CSF sampling, which may have reduced CSF culture sensitivity despite molecular detection. Diagnostic performance metrics are summarized in Table 2.

Blood cultures were obtained within a clinically relevant timeframe around CSF sampling in 17 of the 23 BCID2-positive episodes (73.9%) and were therefore included in the CSF–blood culture comparison. Among these 17 episodes, blood cultures were positive in 13/17 (76.5%) and negative in 4/17 (23.5%). Concordance between CSF BCID2 findings and blood culture results was observed in 12/13 (92.3%) blood culture-positive episodes. In the remaining discordant episode, *C. albicans* was detected by BCID2 on CSF, whereas a coagulase-negative staphylococcus was isolated from blood culture. In the other 6/23 episodes (26.1%), concomitant blood cultures were not obtained within the predefined timeframe and were therefore excluded from the CSF–blood culture comparison.

Complete CSF chemical–physical data were available for 21 of the 23 BCID2-positive samples (Table 3). CSF glucose concentrations were below the pediatric reference range (60–80 mg/dL) in all cases (21/21, 100%), with values < 30 mg/dL observed in 11/21 samples (52.4%). Total protein levels exceeded 100 mg/dL in 15/21 samples (71.4%). Elevated lactate concentrations (>4.5 mmol/L) were observed in 14/21 samples (66.7%), with peak values up to 19.3 mmol/L. Albumin levels were increased (>35 mg/dL) in 16/21 samples (76.2%). White blood cell (WBC) counts exceeded 30 cells/mm^3^ in 16/21 samples (76.2%), ranging from mild pleocytosis to >12,000 cells/mm^3^. Red blood cells (RBCs) were detected in most samples and were occasionally markedly elevated. Macroscopic CSF appearance was abnormal in 17/21 samples (81.0%), including xanthochromia or hematic discoloration and turbidity in 14/21 cases (66.7%). Overall, these CSF macroscopic and cytochemical findings were consistent with suspected CNS infection and provide supportive evidence that BCID2 detections reflect clinically meaningful microbial findings in this selected cohort.

### Cohort Description and Clinical Impact on Antimicrobial Management (Subset-Based Assessment)

This retrospective cohort included pediatric patients with suspected CNS infection in whom BCID2 testing was applied off-label on residual CSF following a negative ME panel result, in the presence of persistent clinical suspicion and abnormal CSF chemical–physical parameters. Clinical scenarios prompting diagnostic escalation were frequently complex and included neurosurgical conditions and/or ventricular devices (e.g., external ventricular drain [EVD], ventriculoperitoneal [VP] shunt, Rickham reservoir), ICU admission, and comorbidities associated with increased infectious risk, such as immunosuppression, malignancy, prematurity, or congenital CNS anomalies.

In this setting, CSF parameter alterations may overlap with post-surgical inflammation or device-related changes; BCID2 was used as an adjunct tool to provide rapid etiological information, including resistance-gene detection, while conventional culture typically required longer turnaround times.

A detailed infectious diseases-driven clinical review was feasible only for the subset of episodes with complete clinical documentation (*n* = 15; Table 4). In this subgroup, the median age was 13 months (IQR 5.5 months–8.0 years; range 15 days–18 years). Comorbidities were present in 12/15 patients (80.0%), predominantly neurologic/neurosurgical conditions (10/15, 66.7%), whereas 3/15 (20.0%) had no reported comorbidity. Within this subset, BCID2 results informed antimicrobial optimization, most commonly through escalation when empiric therapy did not provide adequate coverage and through earlier initiation of targeted antifungal therapy in Candida CNS infections. Culture remained negative in 2/15 episodes despite BCID2 positivity. BCID2 provided microbiological identification within hours, compared with longer and variable times for culture-based identification (commonly ≥24 h and up to 72 h in our dataset when growth occurred; Table 4). Two deaths occurred in this subgroup, both due to septic shock (one associated with *Salmonella* infection and one with ESBL-producing *Escherichia coli*), in the context of relevant perinatal history or delayed presentation; routine laboratory evaluation showed no evidence of immunodeficiency. Given the retrospective design and the absence of a comparator group, no causal inference regarding the diagnostic strategy and clinical outcome can be made.

In the overall BCID2-positive dataset (*n* = 23 episodes), stewardship-oriented antimicrobial management actions recorded after availability of BCID2 results are summarized in Table 5. Escalation was documented in 13/23 episodes (56.5%), discontinuation in 2/23 (8.7%), and confirmation of the empiric regimen in 9/23 (39.1%). No de-escalation events were documented. Two episodes showed no documented antimicrobial management action attributable to BCID2 results. Stewardship-oriented actions were categorized using predefined operational definitions. Because more than one action could be attributed to the same BCID2 result, categories were not mutually exclusive. Percentages were therefore calculated independently using the total number of BCID2-positive diagnostic episodes as the denominator and should not be summed.

## 3. Discussion

This study evaluated the off-label application of the BCID2 panel on CSF samples from pediatric patients with suspected bacterial meningitis and negative ME panel results in the presence of abnormal chemical–physical profiles. The rationale for this diagnostic approach arises from the need to enhance pathogen detection in high-risk clinical contexts where conventional microbiological methods may be limited, particularly in pretreated or immunocompromised patients. Rapid molecular diagnostics are increasingly recognized as an important contributor to reducing time to etiological diagnosis in CNS infections, a setting in which diagnostic delays may adversely affect clinical management and outcomes [10].

In this cohort of 76 ME-negative CSF samples selected on the basis of persistent clinical suspicion and altered biochemical parameters, BCID2 identified pathogens in 23 cases (30.3%). Concordance with conventional culture was observed in 82.6% of BCID2-positive episodes; the remaining 17.4% were BCID2-positive/culture-negative, with findings that were clinically plausible and, in part, supported by contemporaneous cultures from other sites, as detailed below. These findings support the added value of molecular detection in scenarios where traditional microbiology may be compromised by prior antimicrobial exposure or low microbial burden and are consistent with the concept that a negative CSF culture does not exclude infection in selected high-risk or healthcare-associated contexts [6,11].

The diagnostic performance of BCID2 compared with culture, summarized in Table 2, showed a PPA of 100% and an NPA of 93.0%, with an overall concordance of 94.7%. Importantly, discordant BCID2-positive/culture-negative episodes were characterized by CSF profiles consistent with CNS infection, supporting the clinical plausibility of molecular detections in these cases. This observation aligns with previous evidence demonstrating that antimicrobial pretreatment can significantly reduce CSF culture sensitivity in pediatric bacterial meningitis [6,7]. Together, these findings highlight the importance of integrating molecular results with biochemical and clinical data rather than interpreting culture results in isolation, particularly in complex infectious syndromes [10]. Moreover, for two discordant episodes (*Streptococcus* spp. and *S. aureus*), contemporaneous cultures from other clinical specimens yielded concordant organisms (*S. intermedius* from deep pus; *S. aureus* from pus and biopsy), whereas no microbiological confirmation from other anatomical sites was available for the *S. pyogenes* and *E. faecium* episodes. All four discordant episodes occurred while patients were receiving antimicrobial therapy at the time of CSF sampling, which may have reduced CSF culture sensitivity despite molecular detection. Notably, one episode included in the present cohort, previously reported by our group as a single case report, involved bacterial meningitis caused by a non-K1 Escherichia coli strain outside the FilmArray ME panel target list. This prior report is cited here only to provide additional clinical context for that episode and to illustrate the practical implications of limited panel coverage [12].

Empiric antimicrobial regimens at the time of CSF sampling were frequently broad-spectrum, reflecting the high-risk clinical context in which BCID2 testing was applied. Following availability of BCID2 results, antimicrobial management was optimized in a stewardship-oriented manner, most commonly through escalation when the empiric regimen did not provide adequate coverage and through earlier initiation of targeted antifungal therapy in Candida CNS infections. The distribution of stewardship-oriented actions is summarized in Table 5, while episodes with detailed timing data are reported in Table 4. For example, early detection of carbapenemase genes in *P. aeruginosa* supported escalation to active therapy prior to phenotypic confirmation.

These findings are consistent with previously reported limitations of the ME panel. Despite its excellent sensitivity and specificity in clinical validation studies [4,13], the ME panel is restricted to a predefined range of targets and does not include several clinically relevant healthcare-associated or resistant pathogens, such as *P. aeruginosa*, *Acinetobacter* spp., or *Candida* spp. This limitation is particularly relevant in healthcare-associated ventriculitis and meningitis, where the pathogen spectrum is frequently dominated by staphylococci and resistant Gram-negative bacilli and where clinical guidelines continue to emphasize CSF culture as the cornerstone diagnostic test [11]. In this study, the pathogen distribution detected by BCID2, enriched in *Staphylococcus* spp., *P. aeruginosa*, and *Candida* spp., is consistent with a high-risk case mix plausibly including device-associated and nosocomial scenarios, in which ME panel coverage may be insufficient.

Previous reports have described off-label BCID2 use on sterile fluids, including CSF and synovial fluid, suggesting that BCID2 may complement existing molecular diagnostics in selected high-risk cases [7,14]. Our findings support this hypothesis, demonstrating that BCID2 can identify clinically relevant pathogens in ME-negative pediatric meningitis/CNS infection cases when guided by strong clinical and biochemical suspicion. Notably, CSF profiles in BCID2-positive samples frequently showed features consistent with CNS infection, such as hypoglycorrhachia, hyperproteinorrachia, elevated lactate, and pleocytosis (Table 3), further supporting the plausibility of molecular detections even when culture was negative. The frequent presence of RBCs, potentially reflecting traumatic taps or hemorrhagic CSF, underscores the need to interpret cytochemical findings within the broader clinical and procedural context rather than in isolation. Therefore, since BCID2 is validated for positive blood culture bottles, its use on CSF is off-label and results should be interpreted in clinical context and alongside conventional microbiology.

A key added value of BCID2 in this cohort was the early detection of antimicrobial resistance markers, identified in 30.4% of BCID2-positive samples, including *bla*VIM, *van*A/B, and CTX-M. From an antimicrobial stewardship perspective, access to resistance-gene information within hours may support earlier optimization of therapy in critically ill or immunocompromised children, in whom delays may be clinically consequential. This aspect is particularly relevant in pretreated settings, where conventional culture methods may yield false-negative results due to suppression of pathogen growth, limiting timely confirmation of the diagnosis or targeted adjustment of therapy [15].

The clinical relevance of turnaround time was further supported by the subset with detailed timing data (Table 4), in which BCID2 consistently provided earlier microbiological identification than culture-based methods, including in selected episodes where culture remained negative despite BCID2 positivity. This was especially notable in fungal CNS infections, where early detection of *Candida* spp. supported earlier initiation of targeted antifungal therapy. Although clinical outcomes were not assessed prospectively, early initiation of appropriate antimicrobial therapy remains a cornerstone of meningitis management, including healthcare-associated presentations, to reduce the risk of complications [16].

Stewardship-oriented outcomes were evaluated across the broader BCID2-positive dataset (Table 5). Among 23 positive episodes, escalation was documented in 13/23 cases, while no de-escalation events were recorded; discontinuation occurred in 2/23 cases, and confirmation of the empiric regimen in 9/23 cases (categories not mutually exclusive). Two episodes showed no documented antimicrobial-management action attributable to BCID2 results. This distribution is consistent with the clinical selection strategy adopted (ME-negative cases retained because of persistent suspicion and abnormal CSF profiles) in which clinicians may be more likely to intensify or confirm therapy rather than de-escalate. Accordingly, the observed “clinical impact” should be interpreted primarily as decision support for antimicrobial optimization in complex, high-risk scenarios rather than as evidence of a direct causal effect on patient outcomes. In this series, BCID2 most often supported escalation or confirmation of ongoing therapy rather than de-escalation. This pattern is likely explained by the highly selected population, which included children with persistent suspicion of CNS infection after a negative ME panel, and by the off-label nature of BCID2 testing on CSF. In this preliminary real-world setting, clinicians used BCID2 results to support early antimicrobial optimization while awaiting culture and phenotypic antimicrobial susceptibility testing, but they were generally cautious about narrowing therapy solely on the basis of an off-label molecular result in severe CNS infection. Therefore, the absence of de-escalation should not be interpreted as lack of stewardship value; rather, it reflects the case mix and the safety-oriented approach adopted in this high-risk setting. Prospective studies with predefined indications and stewardship endpoints are needed to determine whether BCID2 may also support earlier de-escalation and antimicrobial-sparing strategies.

This study has several strengths. Its pragmatic, real-world design reflects routine practice in a tertiary pediatric hospital with 24/7 syndromic diagnostic availability and multidisciplinary decision-making. Inclusion criteria were clearly defined and consistently applied, focusing on ME-negative CSF samples with persistent clinical suspicion of CNS infection and abnormal chemical–physical parameters, closely mirroring scenarios in which extended molecular testing is most likely to be considered. The exclusive pediatric and neonatal population further strengthens the relevance of these findings, given the vulnerability of this group to neurological sequelae. Integration of biochemical, microbiological, and molecular data also strengthened interpretation of BCID2 results, particularly in complex and culture-negative cases.

Several limitations should be acknowledged. The retrospective and observational design limited the standardized assessment of time-dependent variables and clinical outcomes. Evaluation of stewardship-oriented clinical impact was feasible only when sufficient documentation was available and was therefore reported descriptively, with explicit denominators and non-mutually exclusive categories. In addition, the decision to perform BCID2 testing, although guided by prespecified criteria, relied on clinical judgment and may have introduced selection bias, limiting generalizability to unselected ME-negative cohorts. Although 76 CSF samples were obtained from 63 patients, these samples corresponded to distinct diagnostic episodes rather than serial follow-up specimens from the same infectious episode. Therefore, diagnostic concordance was assessed at the episode/sample level. However, because some patients contributed more than one episode, these estimates should be interpreted descriptively and not as fully independent patient-level diagnostic accuracy measures. Finally, BCID2 is not validated for CSF testing and remains limited to a predefined list of targets; negative results must therefore be interpreted cautiously and always integrated with clinical assessment and conventional microbiology. Interpretation of frequently detected organisms, such as staphylococci, requires careful clinical correlation, particularly in device-associated scenarios.

Beyond analytical performance, this real-world series highlights a diagnostic stewardship principle: in carefully selected scenarios, the appropriateness of a test may depend on clinical context, pretest probability, and multidisciplinary interpretation rather than on label indication alone. When applied within a defined escalation pathway and interpreted alongside CSF biochemistry and conventional microbiology, off-label molecular tools may provide actionable information not otherwise achievable in a timely manner. Prospective studies should formalize indications and stewardship endpoints for such stepwise strategies.

Taken together, BCID2 demonstrated high diagnostic performance and provided substantial added value in ME-negative pediatric patients with strong clinical and biochemical suspicion of meningitis/CNS infection. Its integration into a stepwise diagnostic workflow may represent a valuable adjunct to existing syndromic tools, particularly in healthcare-associated, device-related, and pretreated patients, where early identification of pathogens and resistance markers can support timely antimicrobial optimization. Prospective studies are warranted to validate BCID2 performance on CSF and to define standardized indications, stewardship endpoints, clinical outcomes, and cost-effectiveness [10,11,12,13,14,15,16].

## 4. Materials and Methods

### 4.1. Study Setting and Patient Selection

This retrospective study was conducted at the Microbiology Unit of Bambino Gesù Children’s Hospital (IRCCS), a tertiary pediatric center in Rome, Italy. Between January 2023 and March 2025, all CSF samples from neonates and children with clinical suspicion of bacterial meningitis underwent standard diagnostics, including biochemical analysis, conventional microbiological culture and ME panel testing, which is available 24/7 as part of the routine workflow.

When the ME panel result was negative despite sustained clinical suspicion of central nervous system (CNS) infection, typically after multidisciplinary discussion with an infectious disease specialist and in the presence of abnormal CSF biochemical parameters (e.g., pleocytosis, hypoglycorrhachia, elevated protein or lactate levels), residual CSF aliquots were further analyzed using the BCID2 panel. This use of the BCID2 panel was considered off label. The use of residual CSF avoided additional invasive procedures in a vulnerable pediatric population. All CSF samples analyzed with the BCID2 panel were retrospectively reviewed for inclusion. Eligible cases met the following criteria: ME panel-negative result, strong clinical suspicion of CNS bacterial infection and evidence of abnormal CSF chemical–physical profile. Exclusion criteria included traumatic lumbar puncture without signs of infection and incomplete diagnostic workup. Based on these criteria, 76 CSF samples, corresponding to 76 distinct diagnostic episodes from 63 pediatric patients, were included in the final analysis. In patients who contributed more than one CSF sample, samples represented separate diagnostic episodes rather than serial follow-up specimens from the same infectious episode. Analyses were therefore primarily conducted at the diagnostic episode/sample level unless otherwise specified. A comparative summary of the three diagnostic methods is presented in Table 6.

### 4.2. Molecular Testing: ME and BCID2 Panels

All CSF samples were tested using the ME panel (bioMérieux, Marcy-l’Étoile, France), a multiplex PCR-based system specifically validated for CSF specimens. This panel enables the simultaneous detection of 14 pathogens, including six common bacterial agents (*Escherichia coli* K1, *Haemophilus influenzae*, *Listeria monocytogenes*, *Neisseria meningitidis*, *Streptococcus agalactiae*, and *Streptococcus pneumoniae*), seven viral pathogens and the yeast *Cryptococcus neoformans/gattii*.

In selected ME-negative cases with biochemical alterations suggestive of infection, residual CSF was subsequently tested with the BCID2 panel. The BCID2 panel was performed off-label on residual CSF aliquots remaining after routine diagnostic testing (ME panel and culture) in ME-negative samples selected for persistent clinical suspicion and abnormal chemical–physical parameters, without any additional sampling. The BCID2 panel includes 43 molecular targets, covering a broad range of Gram-positive and Gram-negative bacteria, yeast species, and a comprehensive panel of resistance genes, including those for extended-spectrum β-lactamases (CTX-M), methicillin resistance (*mec*A/C and *MREJ*), vancomycin resistance (*van*A/B), carbapenemases (KPC, NDM, VIM, OXA-48-like, IMP), and colistin resistance (*mcr*-1). A detailed comparison of the microbial targets included in the ME and BCID2 panels is provided in Table 7.

### 4.3. Diagnostic Workflow

**Figure 1:**
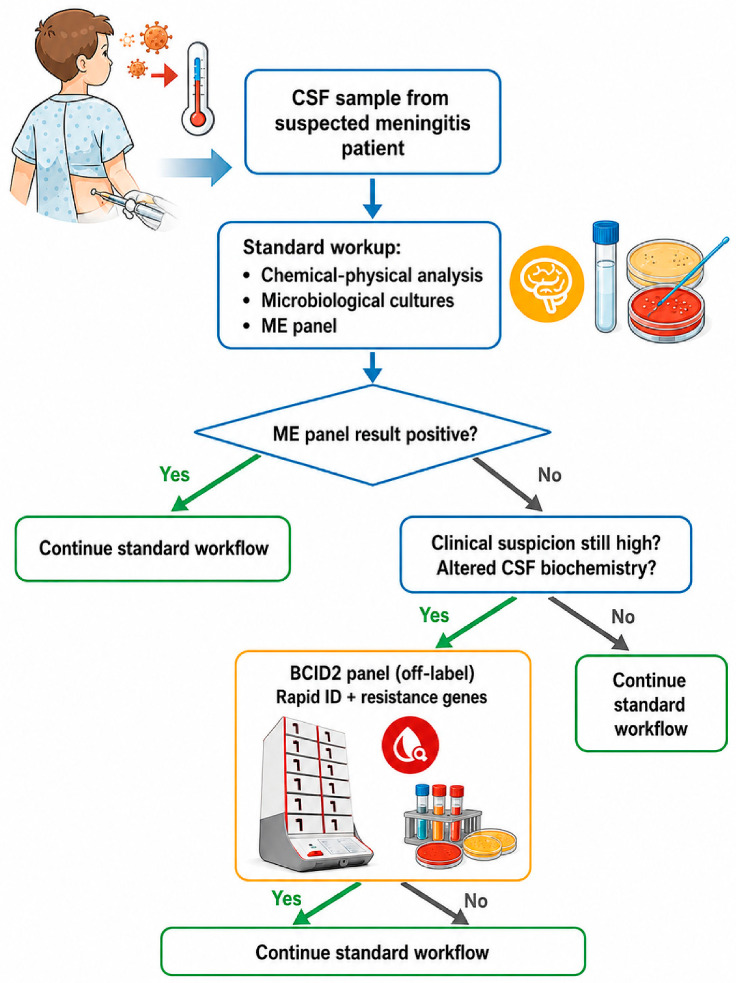
Stepwise diagnostic workflow and escalation pathway for off-label FilmArray BCID2 testing on residual cerebrospinal fluid (CSF) after a negative FilmArray Meningitis/Encephalitis (ME) panel in selected pediatric patients with persistent clinical suspicion of CNS infection and abnormal CSF biochemical parameters.

The diagnostic workflow for suspected CNS infections at our institution integrates conventional and molecular methods and is summarized in Figure 1. Upon clinical suspicion of meningitis or encephalitis, CSF samples are immediately processed for chemical–physical analysis, Gram stain, conventional culture, and multiplex molecular testing using the ME panel. This first-line syndromic approach is performed 24/7 and provides rapid pathogen detection in most routine cases.

If the ME panel returns a positive result, culture confirmation and antimicrobial susceptibility testing (AST) are performed on the same sample. In contrast, when the ME panel is negative, culture remains the standard reference method available, although culture results typically require 24 to 72 h and may be compromised by prior antimicrobial therapy.

In the presence of a negative ME result but persistent clinical suspicion of CNS infection, supported by abnormal CSF biochemical parameters (e.g., pleocytosis, hypoglycorrhachia, elevated protein, or increased lactate), the institutional workflow allows escalation to off-label testing with the BCID2 panel on residual CSF. This second-line decision is taken in collaboration with infectious disease specialists and intensive care physicians, particularly in high-risk or immunocompromised patients. BCID2 testing is performed on residual CSF material remaining after completion of the routine diagnostic work-up. No additional lumbar puncture or device tapping is performed for study purposes, and testing does not modify standard sample collection procedures.

### 4.4. Microbiological and Biochemical Analyses

BCID2 testing was performed by applying the manufacturer’s standard protocol for positive blood cultures directly to residual CSF specimens, without any pre-analytical modification. Results were typically available within 1.5 to 2 h from sample loading. Molecular findings were systematically compared with those obtained by conventional culture.

Culture-positive isolates were further identified at the species level using matrix-assisted laser desorption/ionization time-of-flight mass spectrometry (MALDI-TOF MS; Bruker Daltonics, Bremen, Germany). AST was performed using the broth microdilution method with the MicroScan WalkAway system (Beckman Coulter Inc., Carlsbad, CA, USA). Results were interpreted according to EUCAST clinical breakpoints, applying versions 13.1 to 15.0 depending on the year of testing [17].

For all cases, CSF chemical–physical parameters, including glucose, total protein, albumin, chloride, lactate, white blood cell count (WBC), and red blood cell count (RBC), were retrospectively collected and analyzed. Parameters were interpreted in the context of pediatric reference ranges and used to support the clinical suspicion of CNS infection.

### 4.5. Clinical Data Collection and Assessment of Clinical Impact

Clinical information was retrospectively extracted from electronic medical records and infectious diseases consultation notes, when available. Variables included age group; underlying conditions (e.g., immunosuppression/oncologic disease, prematurity, neurosurgical history); type of CSF sampling or access (lumbar puncture versus ventricular devices such as external ventricular drain [EVD], ventriculoperitoneal [VP] shunt, or Rickham reservoir); intensive care unit (ICU) admission; empiric antibiotic therapy and antimicrobial management following microbiological results. Blood culture results, when available, were also retrieved and compared with CSF BCID2 findings.

Because of the retrospective design and heterogeneous documentation, a complete assessment of clinical impact (including detailed timing of therapeutic modifications and evaluation of empiric regimen appropriateness against the identified pathogen) was feasible only for the subset of episodes. Stewardship-oriented outcomes were therefore reported descriptively for this subset, explicitly indicating denominators for each variable, and included antimicrobial escalation, de-escalation, discontinuation, or confirmation of empiric therapy (categories not mutually exclusive). When available, turnaround times for microbiological identification were also recorded. Because BCID2 was performed in real time on residual CSF as part of clinical care, its clinical impact was assessed retrospectively based on available documentation rather than within a predefined prospective protocol.

Healthcare-associated CNS infection was considered in patients with neurosurgical procedures and/or ventricular devices, in accordance with established definitions and clinical practice guidelines, whereas community-acquired meningitis was considered in patients without neurosurgical devices or recent neurosurgical interventions. Accordingly, findings related to clinical impact should be interpreted as descriptive and supportive of clinical decision-making, rather than as evidence of a causal effect on therapeutic management.

### 4.6. Ethics Statements

This study was conducted in accordance with institutional regulations regarding the off-label use of diagnostic tests and retrospective data analysis. All patient data were fully anonymized, and no procedures were performed outside of routine clinical care.

The authors confirm that all procedures involving human participants were conducted in compliance with the ethical standards of the institutional and national research committees and with the 1964 Helsinki Declaration and its later amendments. Personal data were limited to essential diagnostic information and processed in accordance with Italian privacy legislation (D. Lgs. n.196/2003).

Written study-specific informed consent was not required because of the retrospective design, the use of residual CSF material collected during routine diagnostic care, and the absence of identifiable patient information. Nevertheless, the study was formally approved by the hospital’s Scientific Directorate, as per institutional policy for all research activities. Furthermore, informed consent for the use of anonymized clinical data for diagnostic, therapeutic, and research purposes was obtained from parents or legal guardians at the time of hospitalization.

### 4.7. Statistical Analysis

Analyses were primarily descriptive. Continuous variables were summarized as medians with interquartile ranges (IQR), while categorical variables were reported as absolute numbers and percentages. Diagnostic agreement between the BCID2 panel and conventional culture (reference standard) was evaluated by calculating the Positive Percent Agreement (PPA) and Negative Percent Agreement (NPA). PPA was defined as the proportion of culture-positive samples also positive by BCID2: TP/(TP + FN) × 100. NPA was defined as the proportion of culture-negative samples also negative by BCID2: TN/(TN + FP) × 100, where TP = true positives, FN = false negatives, TN = true negatives, and FP = false positives. No multivariable analyses were performed. Ninety-five percent confidence intervals (95% CIs) for PPA, NPA, overall agreement, and discordant proportions were calculated using the exact binomial method. Diagnostic agreement analyses were performed at the diagnostic episode/sample level, because each CSF sample corresponded to a distinct diagnostic episode with paired BCID2 and culture results. Although some patients contributed more than one diagnostic episode during the study period, these episodes were analyzed independently because they did not represent repeated follow-up testing of the same infection. Accordingly, concordance estimates should be interpreted as descriptive episode-level measures.

## 5. Conclusions

Off-label BCID2 testing on residual CSF provided rapid microbiological identification and resistance-gene information in selected pediatric patients with negative ME panel results but persistent clinical and biochemical suspicion of CNS infection.

These results suggest that BCID2 may have a complementary role as a second-line diagnostic tool within a stepwise workflow for high-risk pediatric CNS infections, particularly in healthcare-associated, device-related, immunocompromised, or pretreated patients, where culture sensitivity may be reduced and rapid resistance-gene detection may support earlier antimicrobial optimization. However, because BCID2 is not validated for CSF testing, its use should remain restricted to carefully selected cases and interpreted through multidisciplinary clinical–microbiological assessment rather than as a standalone diagnostic result.

Prospective studies are now required to validate BCID2 performance on CSF, define standardized indications for its off-label use, and assess its impact on antimicrobial stewardship metrics, clinical outcomes, and cost-effectiveness. More broadly, this experience supports the value of diagnostic stewardship approaches in which rapid molecular tools are integrated into predefined clinical pathways to improve the timeliness and precision of microbiological diagnosis in complex pediatric CNS infections.

## Figures and Tables

**Table 1:** Overview of CSF Sample Analysis and Diagnostic Positivity Rates for ME and Off-label BCID2 Testing.

Parameter	*n* (%)
Total CSF samples analyzed	689
ME-positive samples	129 (18.7%)
ME-negative samples	560 (81.3%)
ME-negative samples tested with BCID2	76
BCID2-positive samples	23 (30.3%)
Culture-positive among BCID2-positive	19 (82.6%)
Culture-negative among BCID2-positive	4 (17.4%)

**Table 2:** Diagnostic performance of off-label BCID2 testing on CSF compared with CSF culture.

Diagnostic Metric	Standard Value, % (95% CI)	Numerator/Denominator
Positive Percent Agreement (PPA)	100.0% (82.4–100.0)	19/19
Negative Percent Agreement (NPA)	93.0% (83.0–98.1)	53/57
Overall agreement	94.7% (87.1–98.5)	72/76
Discordant results	5.3% (1.5–12.9)	4/76

Values refer to CSF samples/diagnostic episodes (*n* = 76). Confidence intervals were calculated using the exact binomial method. Culture was used as the comparator method. Discordant results indicate BCID2-positive/culture-negative samples. Discordant results indicate BCID2-positive/culture-negative samples (*Streptococcus* spp., *Streptococcus pyogenes*, *Staphylococcus aureus*, and *Enterococcus faecium*).

**Table 3:** Summary of Chemical–physical Parameters in BCID2-Positive CSF Samples.

Parameter	Median (IQR)	Pediatric Reference Range
Glucose (mg/dL)	26 (13–48)	60–80
Total proteins (mg/dL)	145 (94–186)	<43 (children < 8 y)
Lactate (mmol/L)	6.62 (4.29–8.06)	1.10–4.40
Albumin (mg/dL)	79.8 (44.2–94.1)	14.00–35.00
WBC (cells/mm^3^)	319 (138–693)	0–30
RBC (cells/mm^3^)	178 (39–938)	Absent

**Table 4:** Clinical, CSF, and microbiological characteristics of the subset with detailed clinical data (*n* = 15).

Patient No.	Age	Relevant Comorbidity	Clinical Syndrome/Diagnosis	BCID2 Result	BCID2 TAT, h:mm	Culture ID TAT, h:mm
1	3 months, 18 days	Cerebral malformation	Rickham reservoir-associated infection	*S. epidermidis*	3:35	40:00
2	13 months	Cerebral venous thrombosis	EVD-associated infection	*C. albicans*	3:23	22:00
3	18 years	Cerebral abscess	EVD-associated infection	*A. baumannii*	1:52	48:00
4	7 months, 13 days	None	Meningitis	*E. coli* (ESBL)	2:00	9:00
5	1 month, 26 days	Prematurity; intraventricular hemorrhage	Rickham-associated infection	*E. faecium* (*van*A/*van*B)	2:00	No growth
6	2 months, 13 days	Hydrocephalus	Rickham-associated infection	*S. capitis*	1:15	26:00
7	9 years, 10 months	None	Meningitis with mastoiditis	*S. pyogenes*	1:24	No growth
8	3 years, 9 months	Hemophagocytic lymphohistiocytosis	Meningitis	*P. aeruginosa* (*bla*VIM)	1:11	48:00
9	8 months, 5 days	Hydrocephalus	VP shunt-associated infection	*C. albicans*	1:18	36:00
10	9 months	Brain arteriovenous malformation	EVD-associated infection	*S. epidermidis*	2:45	47:00
11	3 years, 9 months	Brain tumor	Rickham-associated infection	*S. epidermidis*	1:51	46:00
12	15 years, 3 months	Acute lymphoblastic leukemia (ALL)	Meningitis	*P. aeruginosa* (*bla*VIM)	1:15	47:00
13	15 days	None	Meningitis	*Salmonella* spp.	1:16	13:28
14	8 years	Craniopagus twin	EVD-associated infection	*C. albicans*	1:43	23:00
15	8 years	Craniopagus twin	EVD-associated infection	*C. albicans*	1:48	23:00

CSF, cerebrospinal fluid; EVD, external ventricular drain; VP, ventriculoperitoneal; ESBL, extended-spectrum β-lactamase; TAT, turnaround time. Times represent laboratory turnaround times from sample receipt/acceptance to result availability in the laboratory information system. For BCID2, time refers to panel result availability; for culture, time refers to organism identification. “No growth” indicates no growth on CSF culture.

**Table 5:** Antimicrobial management actions associated with BCID2 results.

Action Category	Operational Definition	n/N (%)
De-escalation	Addition or broadening of antibacterial or antifungal therapy after BCID2 result	0/23 (0.0%)
Escalation	Narrowing of antimicrobial therapy after BCID2 result	13/23 (56.5%)
Discontinuation	Discontinuation of one or more antimicrobial agents after BCID2 result and clinical review	2/23 (8.7%)
Confirmation of empiric therapy	No change because empiric therapy was considered appropriate for the detected pathogen/resistance profile	9/23 (39.1%)
No documented action	No antimicrobial management action clearly attributable to BCID2 in the medical record	2/23 (8.7%)

Actions were derived from chart review and categorized as escalation, de-escalation, discontinuation, confirmation of empiric therapy, or no documented action. Categories were not mutually exclusive; more than one action could be recorded for a given episode.

**Table 6:** Comparative characteristics of diagnostic methods for bacterial meningitis using CSF samples.

Characteristic	Conventional Microbiological Culture	ME Panel	BCID2 Panel (Off-Label)
Regulatory approval for CSF	Approved	Approved	Not validated
Time to result	24–72 h	~1 h	~1 h
Detection of viable organisms required	Yes	No	No
Viral targets included	No	Yes	No
Coverage of multidrug-resistant organisms	Yes	No	Broad spectrum

**Table 7:** Comparison of target organisms between the ME panel and the BCID2 panel.

Pathogen Category	ME Panel (Validated for CSF)	BCID2 Panel (Off-Label Use on CSF)
Gram-Positive Bacteria	*Listeria monocytogenes* *Streptococcus agalactiae* *Streptococcus pneumoniae*	*Enterococcus faecalis* *Enterococcus faecium* *Listeria monocytogenes* *Staphylococcus* spp. *Staphylococcus aureus* *Staphylococcus epidermidis* *Staphylococcus lugdunensis* *Streptococcus* spp. *Streptococcus agalactiae* *Streptococcus pneumoniae* *Streptococcus pyogenes*
Gram-Negative Bacteria	*E. coli* K1 *Haemophilus influenzae* *Neisseria meningitidis*	*Acinetobacter calcoaceticus-baumannii complex* *Bacteroides fragilis* *Enterobacterales* *Enterobacter cloacae complex* *Escherichia coli* *Klebsiella aerogenes* *Klebsiella oxytoca* *Klebsiella pneumoniae group* *Proteus* spp. *Salmonella* spp. *Serratia marcescens* *Haemophilus influenzae* *Neisseria meningitidis* *Pseudomonas aeruginosa* *Stenotrophomonas maltophilia*
Yeast	*Cryptococcus neoformans/gattii*	*Candida albicans* *Candida auris* *Candida glabrata* *Candida krusei* *Candida parapsilosis* *Candida tropicalis* *Cryptococcus neoformans/gattii*
Viruses	Cytomegalovirus (CMV) Enterovirus (EV) Herpes simplex virus 1 (HSV-1) Herpes simplex virus 2 (HSV-2) Human herpesvirus 6 (HHV-6) Human parechovirus (HPeV) Varicella zoster virus (VZV)	Not included
Antimicrobial Resistance Genes	Not included	IMP KPC OXA-48-like NDM VIM *mcr*-1 CTX-M mecA/C mecA/C and *MREJ* vanA/B

## Data Availability

The original contributions presented in this study are included in the article. Further inquiries can be directed to the corresponding author.

## References

[1] van de Beek D., Cabellos C., Dzupova O., Esposito S., Klein M., Kloek A.T., Leib S.L., Mourvillier B., Ostergaard C., Pagliano P. (2016). ESCMID guideline: Diagnosis and treatment of acute bacterial meningitis. Clin. Microbiol. Infect..

[2] World Health Organization (2020). Defeating Meningitis by 2030: Global Roadmap.

[3] Thigpen M.C., Whitney C.G., Messonnier N.E., Zell E.R., Lynfield R., Hadler J.L., Harrison L.H., Farley M.M., Reingold A., Bennett N.M. (2011). Bacterial meningitis in the United States, 1998–2007. N. Engl. J. Med..

[4] Tansarli G.S., Chapin K.C. (2020). Diagnostic test accuracy of the BioFire^®^ FilmArray^®^ meningitis/encephalitis panel: A systematic review and meta-analysis. Clin. Microbiol. Infect..

[5] Trujillo-Gómez J., Tsokani S., Arango-Ferreira C., Atehortúa-Muñoz S., Jimenez-Villegas M.J., Serrano-Tabares C., Veroniki A.A., Florez I.D. (2022). Biofire FilmArray Meningitis/Encephalitis panel for the aetiological diagnosis of central nervous system infections: A systematic review and diagnostic test accuracy meta-analysis. eClinicalMedicine.

[6] Leitner E., Hoenigl M., Wagner B., Krause R., Feierl G., Grisold A.J. (2016). Performance of the FilmArray Blood culture identification panel in positive blood culture bottles and cerebrospinal fluid for the diagnosis of sepsis and meningitis. GMS Infect. Dis..

[7] Pardo J., Klinker K.P., Borgert S.J., Butler B.M., Rand K.H., Iovine N.M. (2014). Detection of Neisseria meningitidis from negative blood cultures and cerebrospinal fluid with the FilmArray blood culture identification panel. J. Clin. Microbiol..

[8] Micó M., Navarro F., de Miniac D., González Y., Brell A., López C., Sánchez-Reus F., Mirelis B., Coll P. (2015). Efficacy of the FilmArray blood culture identification panel for direct molecular diagnosis of infectious diseases from samples other than blood. J. Med. Microbiol..

[9] Michos A., Palili A., Koutouzis E.I., Sandu A., Lykopoulou L., Syriopoulou V.P. (2016). Detection of bacterial pathogens in synovial and pleural fluid with the FilmArray Blood Culture Identification System. IDCases.

[10] Venkatareddy M.P., Upadhya D., Yegneswaran P.P., Varghese A., Pahadasingh S., Prabhu A.N., Saravu K., Shettigar K.S. (2025). Molecular diagnostic methods for rapid diagnosis of central nervous system infections. Front. Med. Technol..

[11] Tunkel A.R., Hasbun R., Bhimraj A., Byers K., Kaplan S.L., Scheld W.M., van de Beek D., Bleck T.P., Garton H.J.L., Zunt J.R. (2017). 2017 Infectious Diseases Society of America’s Clinical Practice Guidelines for Healthcare-Associated Ventriculitis and Meningitis. Clin. Infect. Dis..

[12] Vrenna G., Agosta M., Fox V., Rossitto M., Cortazzo V., Raimondi S., Lucignano B., Onori M., Mancinelli L., Pereyra Boza M.D.C. (2024). Integrating Diagnostic Approaches in Infant Bacterial Meningitis Caused by a Non-K1 Escherichia coli: A Case Report. Antibiotics.

[13] Graf E.H., Farquharson M.V., Cárdenas A.M. (2017). Comparative evaluation of the FilmArray meningitis/encephalitis molecular panel in a pediatric population. Diagn. Microbiol. Infect. Dis..

[14] Guner Ozenen G., Ayhan F.Y., Kacar P., Gulderen M., Yangin Ergon E., Ergun D., Ozbay T., Bayram A., Ozbakır H., Devrim I. (2024). Detection of Pathogens in Cerebrospinal Fluid With the BIOFIRE Blood Culture Identification 2 Panel in Two Neonates With Healthcare-Associated Central Nervous System Infections. Pediatr. Infect. Dis. J..

[15] Nigrovic L.E., Malley R., Macias C.G., Kanegaye J.T., Moro-Sutherland D.M., Schremmer R.D., Schwab S.H., Agrawal D., Mansour K.M., Bennett J.E. (2008). Effect of antibiotic pretreatment on cerebrospinal fluid profiles of children with bacterial meningitis. Pediatrics.

[16] (2024). Meningitis (Bacterial) and Meningococcal Disease: Recognition, Diagnosis and Management.

[17] EUCAST. https://www.eucast.org/clinical_breakpoints/.

